# miRDDCR: a miRNA-based method to comprehensively infer drug-disease causal relationships

**DOI:** 10.1038/s41598-017-15716-8

**Published:** 2017-11-21

**Authors:** Hailin Chen, Zuping Zhang, Wei Peng

**Affiliations:** 1 School of Software, East China Jiaotong University, Nanchang, China; 2 School of Information Science and Engineering, Central South University, Changsha, China; 3 Computer Center of Kunming University of Science and Technology, Kunming, China

## Abstract

Revealing the cause-and-effect mechanism behind drug-disease relationships remains a challenging task. Recent studies suggested that drugs can target microRNAs (miRNAs) and alter their expression levels. In the meanwhile, the inappropriate expression of miRNAs will lead to various diseases. Therefore, targeting specific miRNAs by small-molecule drugs to modulate their activities provides a promising approach to human disease treatment. However, few studies attempt to discover drug-disease causal relationships through the molecular level of miRNAs. Here, we developed a miRNA-based inference method miRDDCR to comprehensively predict drug-disease causal relationships. We first constructed a three-layer drug-miRNA-disease heterogeneous network by combining similarity measurements, existing drug-miRNA associations and miRNA-disease associations. Then, we extended the algorithm of Random Walk to the three-layer heterogeneous network and ranked the potential indications for drugs. Leave-one-out cross-validations and case studies demonstrated that our method miRDDCR can achieve excellent prediction power. Compared with related methods, our causality discovery-based algorithm showed superior prediction ability and highlighted the molecular basis miRNAs, which can be used to assist in the experimental design for drug development and disease treatment. Finally, comprehensively inferred drug-disease causal relationships were released for further studies.

## Introduction

Most drugs achieve their therapeutic functions by binding to specific molecular targets which are relevant to an abnormal state, thereby changing the biochemical and/or biophysical activities of these molecules^1^. Therefore, it is of critical importance to investigate how drugs and diseases form their causal relationships in the molecular level. Many evidences suggest that a drug can act on multiple targets rather than one target^2,3^. More recently, increasing studies revealed that drugs can target microRNAs (miRNAs), which are short (~22 nucleotide) non-coding RNAs, and regulate their expressions. For instance, Rossi *et al*.^4^ detected that the expression levels of 22 miRNAs were altered with the treatment of 5-fluorouracil in human colon cell line.

miRNAs are single-stranded RNAs with post transcriptional regulatory functions. They regulate gene expressions by base pairing to complementary sequences of their target mRNAs^5–7^. Thus, accumulating researches indicated that miRNAs are involved in a broad range of biological processes, such as cellular signaling^8^, proliferation^9,10^ and metabolism^11^. As such, changes in the expression levels of particular miRNAs are related to many kinds of critical diseases. For example, the reduced expression level of let-7 was shown to be associated with lung cancer progression^12^. In addition, miRNAs are suitable to be drug targets as they have several attractive features, such as specific secondary structures and conserved sequences^13^. As a result, restoring miRNA expression levels with small-molecule drugs offers an innovative and promising approach to human disease treatment^13^, which has brought forward to a new research field of miRNAs in pharmacogenomics^14^.

With this understanding, studying drug-disease causal relationships under the perspective of their genetic basis miRNAs could not only help to unveil mechanisms of action of drugs but also advance public health. However, few researches address this question in a systematic view.

Based on chemical descriptors and machine learning algorithms, Jamal *et al*.^15^ created computational models to predict drugs’ biological activities on miRNAs. Jiang *et al*.^16^ applied transcriptional responses to identify associations between drugs and cancer-related miRNAs. Depending on gene expression signatures of bioactive small molecule perturbation and Alzheimer’s disease (AD)-related miRNA regulation, Meng *et al*.^17^ presented a systematic computational approach to constructing a drug-miRNA association network in AD. By integrating features of drugs and miRNAs, Lv *et al*.^18^ proposed an algorithm of Random Walk with Restart (RWR) to infer new associations between small molecules and miRNAs. The above four methods all identify only drug-miRNA associations, but do not comprehensively provide therapeutic potential for drugs.

In the meantime, as miRNAs are highly relevant to multiple complex diseases, many computational efforts^19–33^ have been devoted to detecting potential miRNA-disease associations for understanding the molecular mechanisms of diseases. These methods mainly relay on the assumption that miRNAs tend to show similar dysfunctional evidences for similar disease clusters^34^. Generally, for these researches various features were first integrated for miRN-miRNA and disease-disease similarity calculation. Network-based or machine learning-based algorithms were then developed to rank the most promising disease-related miRNAs or miRNA-related diseass for further biomedical tests. These studies provide reliable guidance for *in vivo* experiment design. However, one limitation lied in these methods is that they could not provide information of disease treatment.

For the above studies, predictions of drug-miRNA associations and miRNA-disease associations were treated separately. We argue that by incorporating information of target miRNAs, we can make more insightful drug-disease causal relationship predictions. Based on the assumption that drugs will form relationships with diseases when they share some significant miRNA partners, Chen *et al*.^35^ applied hyper-geometric tests by combining existing drug-miRNA associations and miRNA-disease associations to predict drug-disease associations. This is the first computational model proposed to infer drug-disease associations, in which the molecular basis miRNAs is explicitly included. Even though a high AUC value could be received, this method is not workable for new drugs whose drug-miRNA associations cannot be obtained.

In this paper, we developed a miRNA-based method to extensively predict drug-disease causal relationships (miRDDCR). Based on two previous researches^18,34^, the proposed method miRDDCR relied on the hypothesis that similar small molecules tend to target similar miRNAs, and finally treat similar diseases. miRDDCR can predict drug-disease relationships in a large scale by combining similarity measurements, existing drug-miRNA associations and miRNA-disease associations. To evaluate the prediction performance of our method, leave-one-out cross validations (LOOCV) were conducted and satisfied AUC values could be received. Compared with existing methods, our method miRDDCR shows superior prediction ability. Moreover, case studies of two drugs demonstrated that our method is powerful in predicting drug-disease causal relationships with a high level of reliability. After validating the usefulness of our method, we used miRDDCR to comprehensively infer drug-disease causal relationships, which we hope will facilitate further drug discovery and disease treatment.

## Results

### Preliminary analysis of datasets used in this manuscript

**Figure 1:**
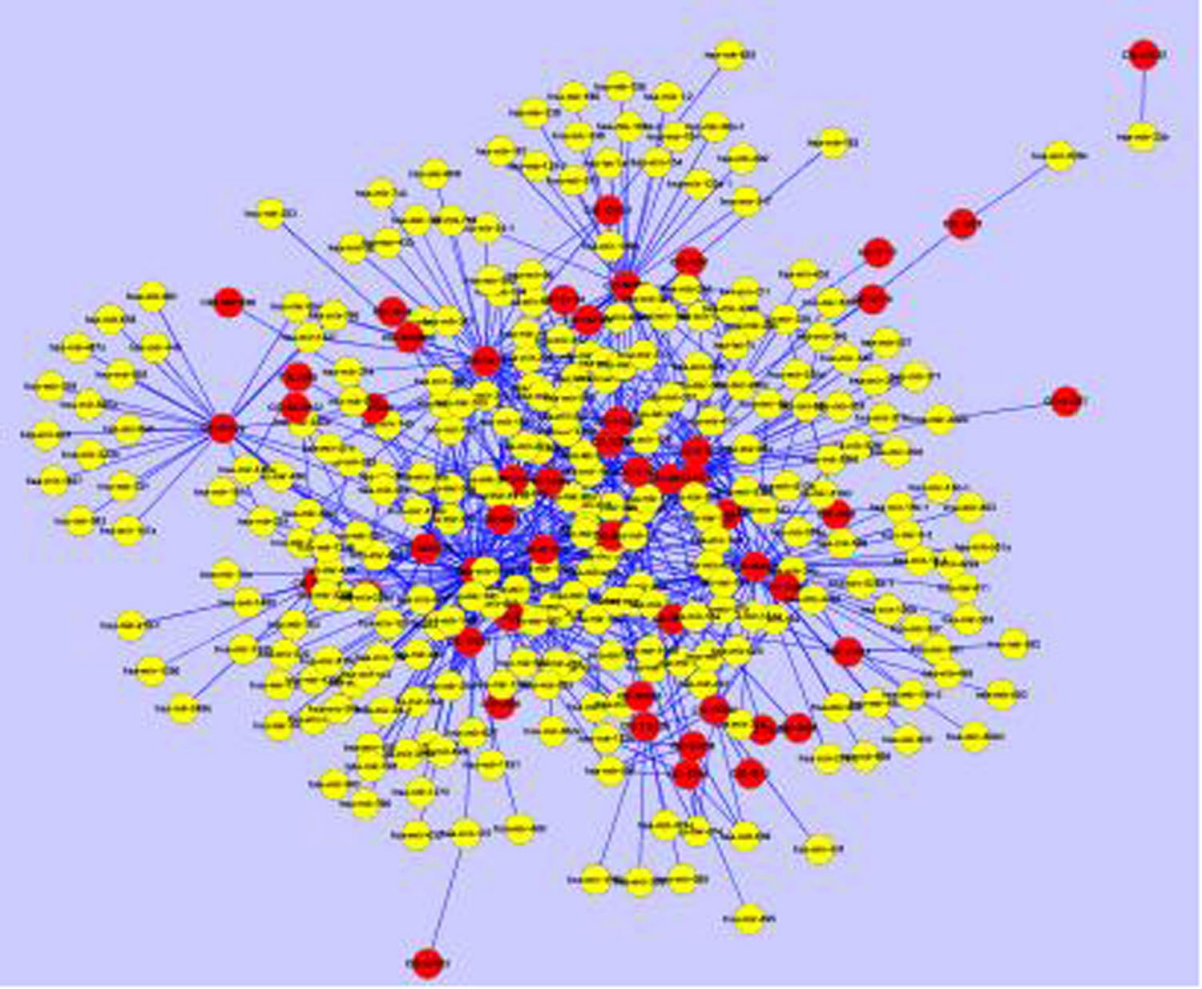
The drug-miRNA bipartite graph. The red circles represent drugs and the pink circles denote miRNAs. This graph was prepared by using the 630 experimentally confirmed drug-miRNA associations.

**Figure 2:**
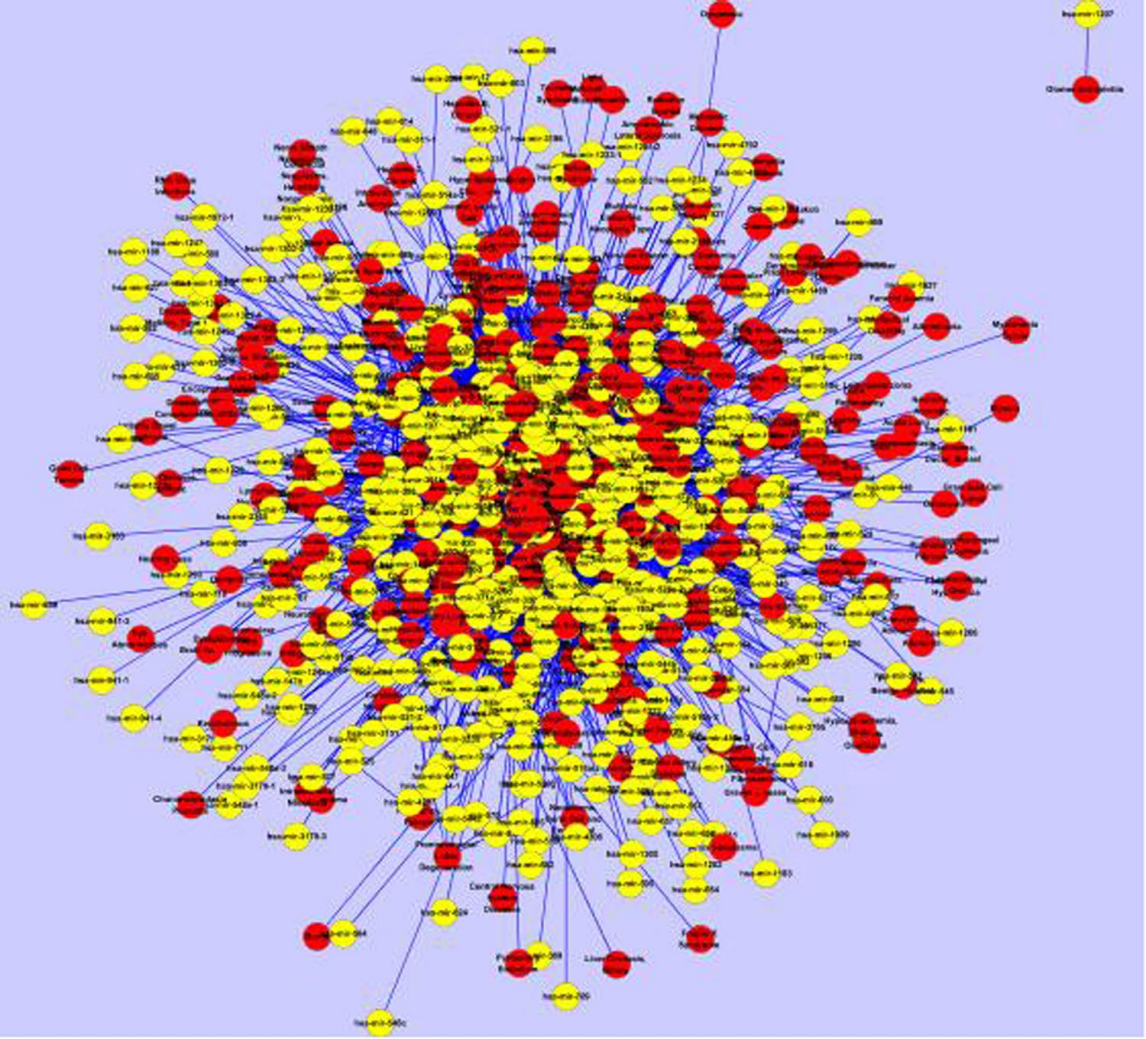
The miRNA-disease bipartite graph. The red circles indicate diseases and the pink circles denote miRNAs. This graph was drawn by using the 6082 known miRNA-disease associations.

The datasets (Supplementary Dataset S1) used in our paper consisted of 831 drugs, 540 miRNAs, 341 diseases, 630 drug-miRNA associations and 6082 miRNA-disease associations. A whole view of the drug-miRNA bipartite graph and the miRNA-disease bipartite graph could be seen in Figs 1 and 2. For the 831 drugs, there were only 51 drugs whose drug-miRNA associations exist. Some statistical analysis of the two bipartite networks was listed at Tables 1 and 2, respectively. We could observe that both the two bipartite graphs were sparse.

**Table 1:** Statistics of the drug-miRNA bipartite graph.

No. of drugs	No. of miRNAs	No. of drug-miRNA associations	Average degree of drugs	Average degree of miRNAs	Sparsity
831	540	630	0.76	1.17	0.0014

**Table 2:** Statistics of the miRNA-disease bipartite graph.

No. of miRNAs	No. of diseases	No. of miRNA-disease associations	Average degree of miRNAs	Average degree of diseases	Sparsity
540	341	6082	11.26	17.84	0.033

### Parameter tuning and performance evaluation of the proposed method miRDDCR

There exist 6 parameters involved in our algorithm. The parameters *α*_1_ and *α*_2_ were decay factors. The other 4 parameters ${l}_{1},{r}_{1},{l}_{2}$ and ${r}_{2}$ were considered as the numbers of maximal iterations of random walks on the bipartite networks. For parameter tuning, we followed ref.^36^ to set ${\alpha }_{1}$ = ${\alpha }_{2}$ = 0.8 and ${l}_{1}={r}_{1}={l}_{2}={r}_{2}=4$.

**Figure 3:**
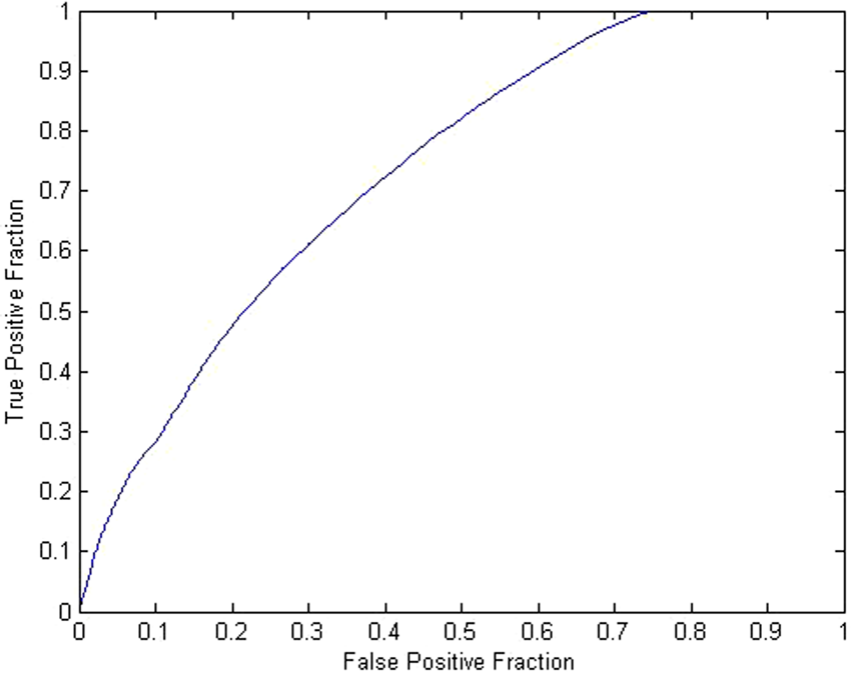
Performance evaluation of miRDDCR in term of ROC curve.

The predicted indication results for the whole 831 drugs were ranked according to the final values received from the algorithm miRDDCR. A bigger value indicated a greater probability that a drug forms a causal relationship with a disease. Experimentally validated drug-disease relationships were extracted from Comparative Toxicogenomics Database (CTD)^37^, DrugBank^38^ and Therapeutic Targets Database (TTD)^39^. We collected 13490 drug-disease relationships which were relevant to our study from the three databases. These confirmed relationships were used as a gold standard dataset for performance evaluation. Taking the known 13490 relationships as the positive instances, a receiver operating characteristics (ROC) curve (see Fig. 3) was drawn by calculating true positive fraction (TPR, sensitivity) and false positive fraction (FPR, 1-specificity) at different cutoffs. Finally, a value of area under curve (AUC) of 0.7334 was received.

**Figure 4:**
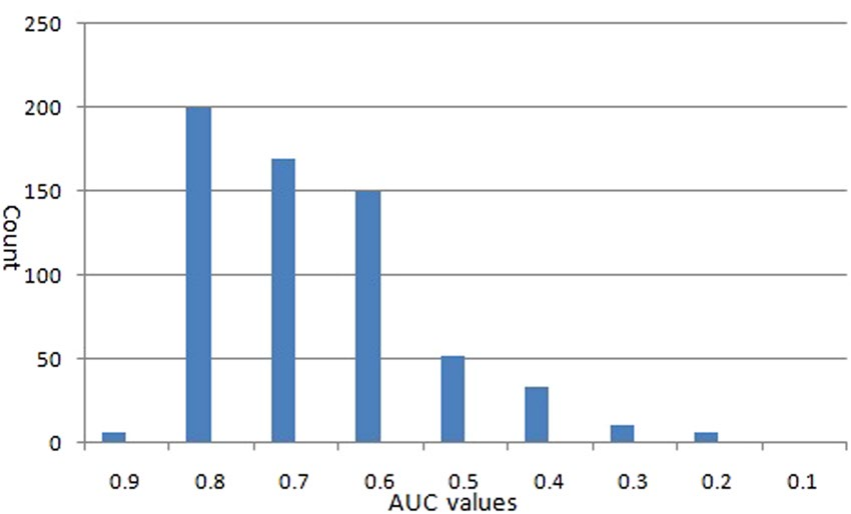
The distribution of AUC values received by leave-one-drug-out cross-validations (LOOCV) for the 630 drugs.

Furthermore, we used leave-one-drug-out cross-validations (LOOCV) to evaluate the performance of miRDDCR in predicting drug-disease causal relationships. For the whole 831 drugs, each drug was considered as a test drug once and all its drug-miRNA association information was removed. The remaining 830 drugs were taken as the training dataset. For the 831 drugs, there existed 201 drugs whose experimentally confirmed drug-disease relationships were not available in the gold standard dataset. Therefore, we could not calculate their AUC values. For the remaining 630 drugs, the distribution of their AUC values, when leave-one-drug-out cross validations were implemented, could be seen in Fig. 4 with an average AUC value of 0.717. Meanwhile, for about 60% (376/630) of the 630 drugs, we received higher AUC values than 0.7, with the highest AUC value of 0.985 for the drug CID:3108 (*Dipyridamole*).

### Comparison with existing methods

Until recently, efforts made on causality discovery-based methods for drug-disease relationship predictions were rare. The most related study to ours is the inference model introduced in ref.^35^, in which drug-disease causality relationship predictions were only based on known drug-miRNA associations and miRNA-disease associations. For novel drugs, whose drug-miRNA associations were not available, their indications could not be inferred by this method. Our method miRDDCR overcame this drawback by taking advantage of similarity measurements. In the leave-one-drug-out cross-validation section, we considered each drug as a novel drug and reliable prediction ability could be obtained.

With the accumulation of biochemical data, several algorithms^40–43^ have been put forward to predict potential drug–disease relationships. They first integrated multiple types of features to construct drug-drug and disease-disease similarity metrics. Depending on the similarity values and experimentally verified drug-disease relationships, machine learning-based or network-based methods were then developed to predict new indications for drugs. Different sources of information prevented a direct comparison between these methods and miRDDCR. Generally, these methods relayed heavily on known drug-disease relationships for new drug-disease association prediction. Obviously, experimentally confirmed drug-disease associations are scarce. The sparsity of data preparation might influence prediction accuracy. Our method did not need known drug-disease associations for prediction. Moreover, these methods, unlike our method miRDDCR, provided no molecular hints to help design experiments to test and confirm the results. Our method made full use of the information of target miRNAs and provided valuable resource for drug design and disease treatment.

### Case studies

In this experimental scenario, case studies of the two drugs, CID:33887 (*Almitrine*) and CID:137 (*Aminolevulinic acid*), were analyzed for further evaluation of the ability of our method miRDDCR to predict potential drug-disease causal relationships.

For the drug CID:33887 (*Almitrine*), there was no target miRNA information in the prepared drug-miRNA dataset. After the first round of bi-random walks in miRDDCR on the drug-miRNA bipartite network, potential target miRNAs were ranked, in which the top 5 miRNAs were hsa-mir-21, hsa-mir-27b, hsa-mir-23a, hsa-mir-27a and hsa-mir-155. With the second round of bi-random walks on the miRNA-disease bipartite network, the indications of the drug *Almitrine* could be received. The top 10 predicted diseases were breast neoplasms, hepatocellular carcinoma, stomach neoplasms, colorectal neoplasms, melanoma, lung neoplasms, neoplasms, ovarian neoplasms, heart failure and prostatic neoplasms. The 9^th^ (9/341) disease heart failure was the only indication available for the drug in the gold standard dataset and we successfully predicted this relationship with a high rank. It should be noted that 4 (hsa-mir-21, hsa-mir-23a, hsa-mir-27a and hsa-mir-155) out of the top 5 target miRNAs, when expressed abnormally, were involved in the development of heart failure^44^.

For the drug CID:137 (*Aminolevulinic acid*), its potential indications were ranked according to the scores received by our algorithm miRDDCR. We selected the top 5, top 10, top 20, top 30 and top 40 predicted diseases and found that there were 5, 9, 17, 21 and 23 results supported by the gold standard dataset, respectively. We took the top 1 predicted disease breast neoplasms as an example to explain the inference ability of our algorithm. For the drug CID:137 (*Aminolevulinic acid*), there was no target miRNA information in the 630 drug-miRNA associations. After the first round of random walk, we chose the top 10 predicted target miRNAs and discovered that the 3rd target (hsa-mir-450a-2), the 5th target (hsa-mir-23a), the 6th target (hsa-mir-29b-2), the 7th target (hsa-mir-320b-1) and the 10th target (hsa-mir-375) were associated with the predicted disease breast neoplasms^44^.

As currently confirmed drug-disease associations were not complete, we think the other predicted diseases with high ranks could be potential indications for the drugs.

### Comprehensive drug-disease causal relationship predictions

After verifying the prediction power of our method by cross-validations and case studies, all the known associations were used as training data to comprehensively predict potential drug-disease causal relationships. Moreover, as causal factors in the molecular level, the top 20 predicted target miRNAs for each drug (Supplementary Dataset S2 online) was available for further experiment tests. For each of the 831 drugs, we published the top 50 predicted candidate indications for future studies. The full list of the whole inferred relationships can be obtained from the Supplementary Dataset S3 online. To be more accurate, we suggested taking the prediction results for the 376 drugs, whose AUC values were greater than 0.7 in leave-one-out cross validations, into consideration.

## Discussion

Exploring the molecular mechanism behind the curative effects of drugs is crucial for drug development and disease treatment. Recent researches suggested that drugs can achieve their therapeutic functions by targeting miRNAs because the abnormal expression of miRNAs could lead to a lot of complex diseases and drugs can bind miRNAs to restore their expression levels. As a relatively new discipline in biomedical research, our understanding of drug-targeted miRNAs and miRNA-related diseases is lacking. Furthermore, few studies attempted to systematically discover drug-disease causal relationships through the target miRNAs.

In this paper, we developed an approach miRDDCR to revealing the causal relationships between drugs and diseases under their molecular basis miRNAs. Our method makes full use of similarity measurements, known drug-miRNA and miRNA-disease associations to infer indications for drugs. As current drug-miRNA and miRNA-disease associations are insufficient, two rounds of bi-random walks are implemented to reveal the hidden associations in the drug-miRNA-disease heterogeneous network. We have applied our method to real datasets and the results showed that our method could successfully discover the causality underlying drugs and diseases. Compared with other algorithms, our method provided a straightforward strategy for causality discovery and showed superior prediction performance.

As similarity values provide a vital role in the prediction procedure, we need to further investigate the types of features collected for similarity measurements. Meanwhile, the predicted drug-disease relationships might be biased as the numbers of known drug-miRNA associations and miRNA-disease associations were rare. We expect the prediction power of our method can be improved by integrating more experimentally confirmed drug-miRNA and miRNA-disease associations.

It should be noted that the mechanism of action of drugs has not been completely investigated. The most recent studies indicated that drugs may also target other non-coding RNAs, including circular RNAs, long non-coding RNAs and Piwi-interacting RNAs (see ref.^45^ for more details). At the same time, complex diseases are multi-factor driven. For example, the study conducted by Wang *et al*.^46^ suggested that DNA mutations and other genomic alterations, which were valuable biomarkers and could be used to construct disease hallmark networks, provided significant roles in cancer clonal evolution and associated clinical phenotypes. Therefore, more relevant biochemical information is needed for deepening our knowledge of drug-disease causal relationships, even though our study provided a feasible strategy for discovering the causality.

## Methods

### Datasets

The 831 drugs used in our manuscript were downloaded from ref.^18^. Similar to ref.^18^, the pairwise similarity values of those drugs were calculated by integrating information of chemical structures^47^, functional consistency^48^ and side effects^40^. The integrated similarity ${S}_{{\rm{d}}}$ was defined as:

1 $${S}_{{\rm{d}}}=({S}_{d}^{c}+{S}_{d}^{f}+{S}_{d}^{s})/3$$

Here, ${S}_{d}^{c}$, ${S}_{d}^{f}$ and ${S}_{d}^{s}$ denoted the similarity measurements based on chemical structures, functional consistency and side effects, respectively.

The 540 miRNAs employed in this paper were also collected from ref.^18^. Similar to ref.^18^, the pairwise similarity ${S}_{{\rm{miR}}}$ of those miRNAs was based on functional consistency^48^.

We obtained 341 diseases and their pairwise similarity measurements ${S}_{{\rm{p}}}$ from ref.^26^. The similarity ${S}_{{\rm{p}}}$ was calculated by incorporating disease semantic similarity and disease functional similarity.

Experimentally validated drug-miRNA associations and miRNA-disease associations were selected from the latest versions of SM2miR^49^ and HMDD^44^, respectively. For drug-miRNA association retrieval, we restricted the species to Homo sapiens. After removing duplicate records stored in the databases of SM2MiR and HMDD, we finally received 630 drug-miRNA associations and 6082 miRNA-disease associations.

### Method Description

We defined the drugs, miRNAs and diseases as $D=\{{d}_{1},{d}_{2},\mathrm{..}.,{d}_{i}\}$, $miR=\{mi{R}_{1},mi{R}_{2},\ldots ,mi{R}_{j}\}$ and $P=\{{p}_{1},{p}_{2},\ldots ,{p}_{k}\}$. Experimentally confirmed drug-miRNA associations were modeled as a bipartite graph ${G}1=\{V1,E1\}$, where $V1=\{D,miR\}$ and $E1=\{{a}_{ij}\,:\,{d}_{i}\in D,mi{R}_{j}\in miR\}$. For $E1$, its values were 1 or 0 which indicated the presence or absence of each association. Similarly, existing miRNA-disease associations were considered as another bipartite graph $G2=\{V2,E2\}$, where $V2=\{miR,P\}$ and $E2=\{{r}_{mn}\,:\,mi{R}_{m}\in miR,{p}_{n}\in P\}$. For $E2$, its values were also 1 or 0 which represented the presence or absence of each association.

**Figure 5:**
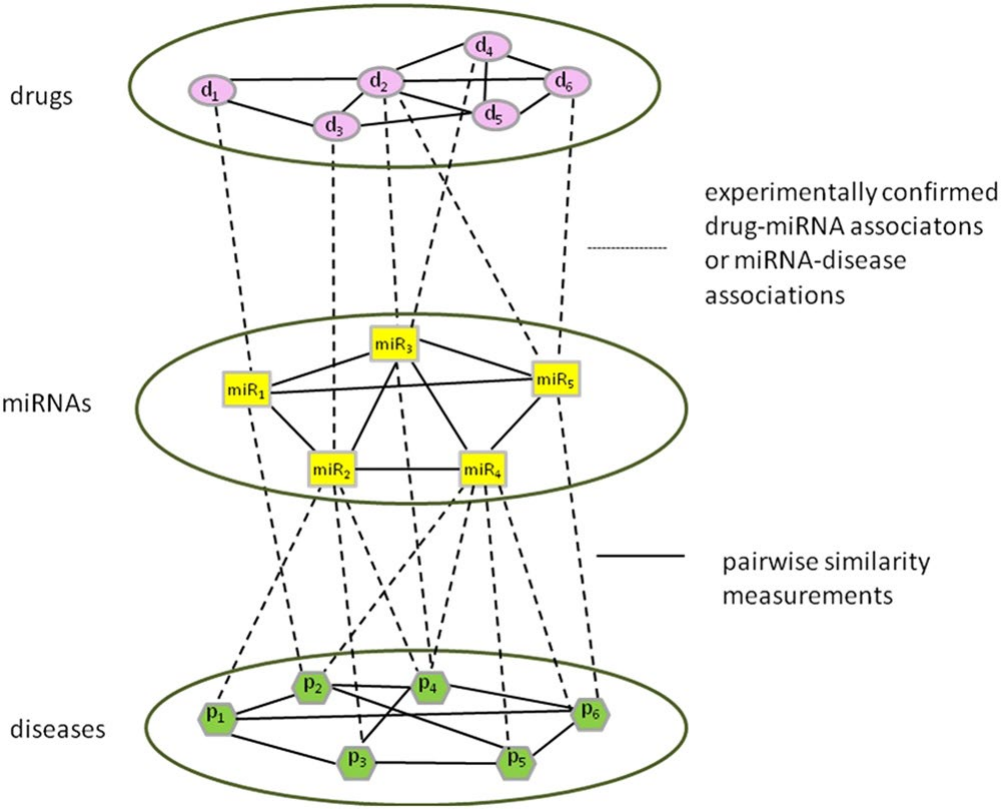
A three-layer drug–miRNA-disease heterogeneous network.

We connected the drug-miRNA bipartite graph and the miRNA-disease bipartite graph together to construct a three-layer drug-miRNA-disease heterogeneous network. For each layer, an edge was drawn between two nodes when the similarity of the two nodes was bigger than 0. The weight of the edge was set to be the similarity value. An example of the heterogeneous network was illustrated in Fig. 5. The objective of this research is to predict drug-disease causal relationships based on the three-layer heterogeneous network.

Previously, random walk^36,50–53^ has been widely used in bipartite graphs for bilateral association prediction in bioinformatics. For example, bi-random walk (BiRW)^36^ was successfully applied on both gene network and phenotype network simultaneously, with an averaged output from the two networks in each step, to infer potential gene-phenotype associations. Inspired by the successful application of BiRW, we extended the algorithm to the three-layer drug–miRNA-disease heterogeneous network to predict potential drug-disease causal relationships.

**Figure 6:**
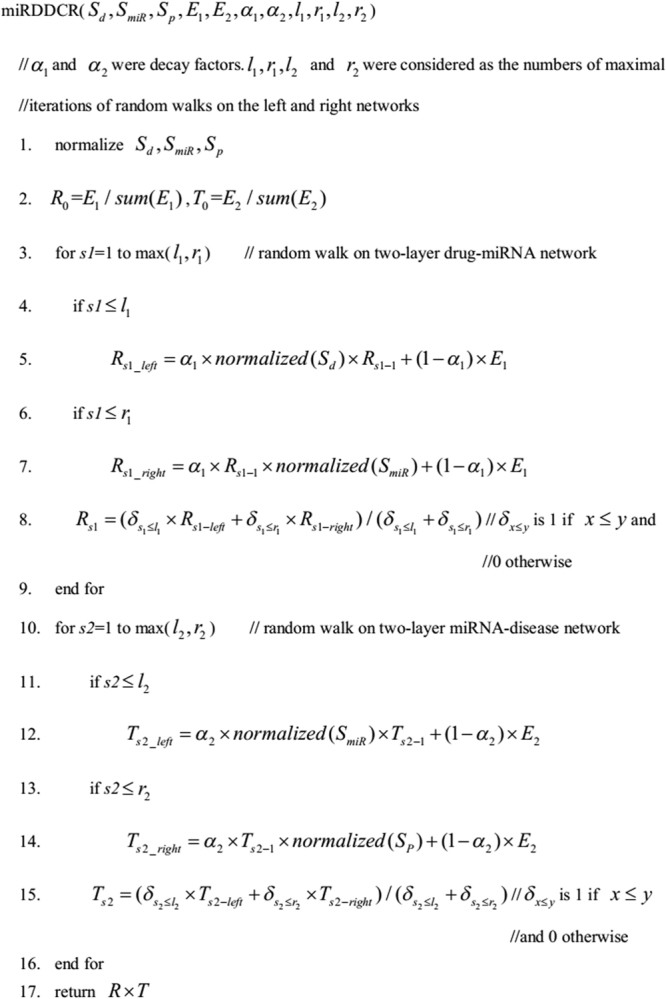
Description of workflows of the algorithm miRDDCR.

In this study, a three-layer heterogeneous network, including a layer of causal factors of miRNAs, was constructed and the drug-disease causal relationship prediction process mainly included three steps. First, bi-random walks were applied on the two-layer drug-miRNA network. Second, another round of bi-random walks was established on the two-layer miRNA-disease network. Finally, the prediction results were received by combining the outcomes from the two previous steps. The whole pipeline of the algorithm miRDDCR could be outlined in Fig. 6.

## Electronic supplementary material

Supplementary Dataset 1

Supplementary Dataset 2

Supplementary Dataset 3
